# Missed gallstones in the abdominal wall: complication of a laparoscopic cholecystectomy

**DOI:** 10.11604/pamj.2020.37.381.27368

**Published:** 2020-12-29

**Authors:** Sofia Frade, Sofia Carrelha, Nuno Monteiro, Luís Moniz, Helder Viegas

**Affiliations:** 1 Department of General Surgery, Centro Hospitalar Universitário de Lisboa Central, Lisbon, Portugal

**Keywords:** Laparoscopic cholecystectomy, gallbladder perforation, gallstone

## Abstract

Laparoscopic cholecystectomy, like any invasive procedure, is associated with complications. One of them often ignored despite its frequency, as the results of the low morbidity rate is stone spillage. We present a case of a 38 years male, with obesity; that underwent laparoscopic cholecystectomy for an acute cholecystitis. During surgery, gallbladder perforation occurred with stone spillage. An attempt was made for recovery of all stones. However, one month after surgery, the patient complained of abdominal pain in the upper right quadrant and an abscess of the deep abdominal wall was found caused by a missed gallstone. Although the definitive treatment was not immediate, an attempt at antibiotic therapy was made, unsuccessfully. Afterwards, this patient underwent gallstone extraction and removal of foreign body granuloma with complete resolution of the clinical condition.

## Introduction

Advances in surgical technique bring challenges beyond the technical aspects. These include new complications that must be in mind. Currently, laparoscopic cholecystectomy is the technique considered gold standard for the treatment of symptomatic gallstones, and one of the procedures most frequently performed by general surgeons. Despite being considered a safe procedure, it has intra-operative complications: gallbladder perforation (10-40%) and stone spillage (6-30%) are two of the most frequent ones [1]. Although initially thought that stone loss was harmless, it is now known to be associated with intra-abdominal complications, associated morbidity and mortality (0.04-19%) [2], despite the evidence that the majority of cases are asymptomatic [3]. We present a case of an abdominal wall abscess a year after laparoscopic cholecystectomy, demonstrating the importance of minimizing gallstone loss.

## Patient and observation

A 38-years-old male, with known history of hypertension, obesity and gallstones, presented in the emergency room with abdominal pain in the upper right quadrant that radiated to the right shoulder. After clinical, laboratory and ultrasound evaluation, the diagnosis of acute cholecystitis was established. He was admitted for a laparoscopic cholecystectomy. Intraoperatively, an acute gangrenous cholecystitis with a contained perforation was found. Due to the marked inflammation, dissection of the hilum and the gallbladder was laborious and gallstones leaked out of the gallbladder. This was identified and an abundant peritoneal lavage was performed as well as an attempt to remove all leaked gallstones. No more intra or postoperative complications were recorded, and the patient was discharged on the 5^th^ postoperative day after antibiotic therapy with Piperacillin/Tazobactam, completely asymptomatic. In a follow-up appointment, 1 month after surgery, the patient reported new onset of abdominal pain, at the upper right quadrant, with progressive worsening. Upon observation, he was sweaty, with pain at the superficial and deep palpation of the right hypochondrium. For that reason, he was referred to the emergency room.

**Figure 1:**
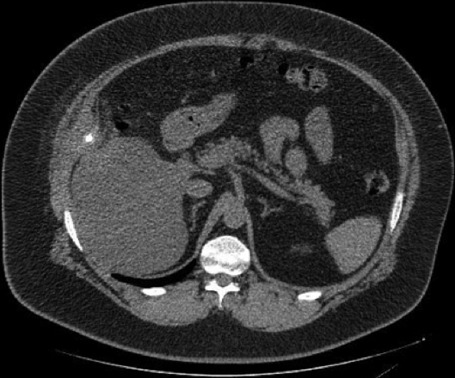
inflammatory reaction after 1 month surgery

**Figure 2:**
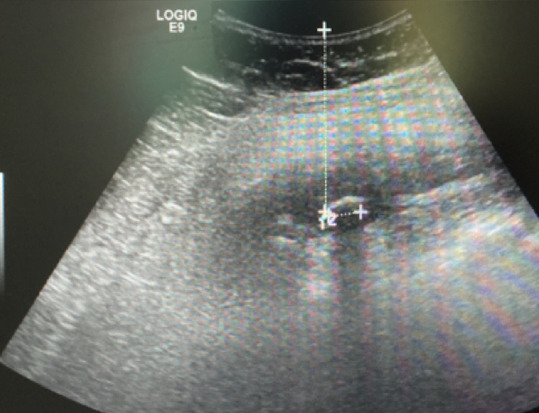
ultrasound images of the lost gallstone

Due to the recent history of surgery, it was decided to perform laboratory and imaging investigation. The blood work showed leukocytosis without neutrophilia and elevated PCR. The computerized tomography (CT) scan revealed: “in the right hypochondrium, at the transverse and internal oblique muscles, a marked thickening with 69x31x26 mm, with a poorly defined margin and with densification of the surrounding fat, and an image of hyper-attenuation with 9mm” (Figure 1). An ultrasound was made to clarify these findings: “a pericentrimetric calcification is observed in the abdominal wall, about 6 cm deep from the cutaneous surface, with adjacent pseudonodular densification”. These findings were compatible with a migrated stone and small surrounding collection (Figure 2). The patient was discharged with the indication for antibiotics therapy (amoxicillin + clavulanic acid) and analgesia. The complaints disappeared and it was decided to keep an expectant attitude. The patient maintained regular follow-up and reported intermittent pain in the referred region but not disabling. For that reason, elective removal of the gallstone was proposed. Before elective surgery, he returned to the emergency department with severe abdominal pain associated with fever and an upper right abdominal tumefaction. Abdominal exam showed a hard, painful mass with inflammatory signs on the right hypochondrium.

**Figure 3:**
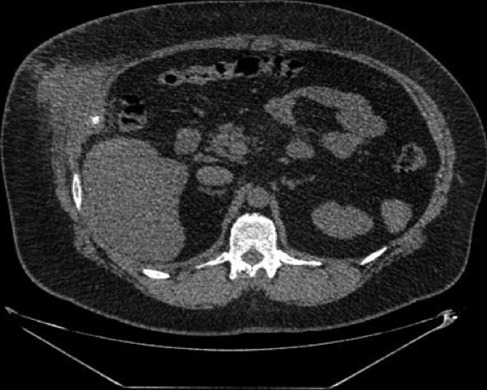
abdominal wall abscess surrounding the lost gallstone

**Figure 4:**
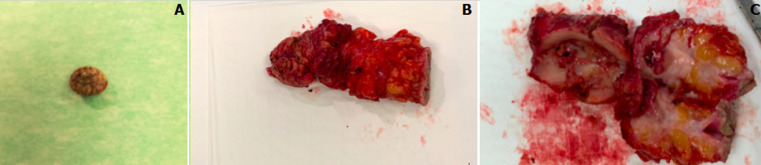
A) lost gallstone capture in the abdominal wall; B) foreign body granuloma removed in surgery; C) open foreign body

Laboratory work revealed leukocytosis without neutrophilia and a new ultrasound and CT scan showed thickening of the abdominal wall, that echographically translated a hypoechoic formation of 6x5 cm and may correspond to an abscess and/or phlegm with possible liquefaction. Also, a prominent external anterior parietal thickening measuring 8x6 cm, with two deep calcifications of 10 and 8 mm, one most likely a chondral alteration and the other a possible gallstone” (Figure 3). An abscess of the abdominal wall due to a foreign body (gallstone) was diagnosed. The patient completed one week of antibiotics and underwent surgery, under general anesthesia. At that time, there was no evidence of abscess on the abdominal wall and a foreign body granuloma was found occupying the entire abdominal wall, with a pigmented gallstone in pre-peritoneal position (Figure 4). So, extraction of the gallstone and the respective foreign body granuloma was performed, which was later confirmed through histopathological examination. Post-operative period was without complications, with progressive improvement of symptoms. The patient was followed for a year, remaining asymptomatic throughout this entire period, without other manifestations due to lost gallstones.

## Discussion

It is well known and established in the scientific community that laparoscopic cholecystectomy is the surgical treatment of choice for the treatment of symptomatic gallstones. It is associated with lower pain in the postoperative period, decreased length of stay, lower perioperative morbidity and mortality rates, and it allows the patient to return to their daily life activities faster [4]. However, like any surgical procedure, it is not without risks, and we begin to see different complications than those described in open cholecystectomy, such as: bile duct and vascular lesions and intraoperative spillage of stones. The latter is often ignored despite its frequency, since it is associated with a small percentage of complicated cases and can be unnoticed for many years. The average time for symptoms to appear is 10.4 months, with cases described in the literature ranging from 10 days to 20 years, postoperatively [5]. The spillage of stones can occur on several occasions during surgery: in the dissection of the gallbladder from its bed (more frequent - 75%), with excessive traction of the gallbladder with a grasper or during its extraction through the port (25%) [6].

In open cholecystectomy it is relatively easy to remove lost gallstones from the abdominal cavity, through irrigation and aspiration, but it becomes more complex in the laparoscopic approach [7]. The stones after entering the peritoneum can migrate into the entire abdominal cavity, the factors that lead to this are the pneumoperitoneum induced for surgery and the peritoneal irritation itself [8]. There are risk factors associated with this complication: male gender, older age, obesity, the complexity of the surgery (acute inflammation), the presence of adhesions, surgeon experience, preoperative pain more than 96 hours and palpable gallbladder in the preoperative period [8]. In the case present above, the patient had 3 risk factors: male, obesity and presence of cholecystitis. As stated earlier, only a small percentage of patients develop complications after spillage of stones (0.08-0.3%). One of the factors that makes most patients asymptomatic is the fact that peritoneal gallstones create an inflammatory process that can lead to partial or complete reabsorption of the stone [9]. The predisposing factors were studied to understand who were the patients with more risk to develop these complications, and those are pointed out in Table 1.

**Table 1:** risk factors for complication after gallstone spillage

Older age
Male sex
Acute cholecystitis
Spillage of pigment stones
Number of stones (>15)
Size of the stone (>1,5 cm)
Perihepatic localization of lost stones
Adapt from Brockmann JG. Complications due to gallstones lost during laparoscopic cholecystectomy. Surg Endosc. 2002; 16:1226-32

Remembering our patient, he had 4 of these risk factors (gender, cholecystitis, pigmented stone and perihepatic position). Contrary to what happens in sterile stones (in which the inflammatory process to the foreign body causes granulomas) [10], pigmented stones have bacterial contamination in 83% of the cases. Also, it is thought that the stones around the liver escape a process of intra-abdominal “clearing" attributed to the great omentum and the intestinal immune system [5]. These two causes can explain the abscess formation in the case presented. On the other hand, the presence of a foreign body naturally explains the formation of a large granuloma. There is a wide spectrum of complications with the existence of gallstones in the abdominal cavity (Table 2). However, the most common is abscess formation (60% of the cases) with *Escherichia coli* being the most common pathogen [2, 8]. The diagnosis of gallstone abscess is usually delayed and can be a challenge, normally only detected if there is a high clinical suspicion. Surgeons have to think in this possibility exploring the surgical history of the patient, and in the event of a previous cholecystectomy, they should inquire the possibility of stone spillage during the procedure. The image studies such as abdominal ultrasound, computed tomography and magnetic resonance imaging, combine or not, can help in the diagnosis [4]. In our patient, the degree of suspicion of postoperative complication was high since it was an immediate postoperative period, so diagnostic tests were helpful diagnosing an abscess of the abdominal wall reactive to a foreign body (gallstone).

**Table 2:** possible complications after gallstone spillage

Infection		Cutaneous complication	Mechanical	Migration to other systems
**Local**	**Distant**	Sinus formation	Intestinal obstruction	Chest: empyema, cholelithoptysis
Liver abscess	**Retroperitoneal abscess**	Port site infection	Lodgement in distant hernial sacs	Urinary tract: excretion, haematuria
Subhepatic abscess	Loin abscess	Granuloma formation	Dyspareunia, tenesmus (pelvic migration)	Systemic
**Retrohepatic abscess**	Pelvic abscess	Colocutaneous fistula		Septicaemia
Intra-abdominal abscess				

Adapt from Sathesh-Kumar T. Spilled gallstones during laparoscopic cholecystectomy: a review of the literature. Postgrad Med J. 2004; 80:77-9

The treatment of these abscesses depends on their location and can include antibiotics, intervention radiology procedures or surgery. In our case we opted for antibiotics and posterior removal of gallstone + granuloma. In retrospective, we should have moved on to definitive surgery as soon as the diagnosis was established and not maintain an expectant attitude. In order to minimize these complications, it is necessary to focus on prevention. That being said, a careful dissection and removal of the gallbladder is essential to prevent gallbladder rupture. In case this is not possible, efforts must be made to minimize spillage, trying to close the gallbladder with the grasp or with an endoclip. Proper irrigation and aspiration should be done and in case of stone spilling, surgeons must attempt to recollect all of them and extract them in an endobag [3]. If necessary, it is more advantageous to increase the dimension of the port for extraction than to risk spillage of gallstones. Most recent studies recognize that there is no need to convert laparoscopic surgery to try to remove gallstones. In our case, the loss of gallstones was noted intraoperatively and every effort was made to remove all of them. However, one of the stones went unnoticed and came the clear cause of this rare and unwanted complication.

## Conclusion

After the presentation of this clinical report and the literature review, we can conclude that despite its low incidence, lost gallstones can lead to a large number of complications and it is essential to maintain high suspicion when we are observing a cholecystectomized patient with abdominal pain. Imagiologic studies can help to establish the correct diagnosis, and it is essential to prevent spillage during surgery and if it occurs, efforts must be made to remove all lost gallstones. The treatment will depend on the localization of the abscess, in this case, a first approach with antibiotic therapy was attempted, however, it was necessary to re-operate the patient in order to remove the lost gallstone.
